# *Bryophyllum pinnatum* enhances the inhibitory effect of atosiban and nifedipine on human myometrial contractility: an in vitro study

**DOI:** 10.1186/s12906-019-2711-5

**Published:** 2019-11-04

**Authors:** S. Santos, C. Haslinger, M. Mennet, U. von Mandach, M. Hamburger, A. P. Simões-Wüst

**Affiliations:** 1 Department of Obstetrics, University Hospital Zurich, Schmelzbergstrasse 12/PF 125, 8091 Zurich, Switzerland; 2 Division of Pharmaceutical Biology, University of Basel, Basel, Switzerland; 3 Weleda AG, Arlesheim, Switzerland

**Keywords:** *Bryophyllum pinnatum*, Atosiban, Nifedipine, Preterm, Myometrium, Contractility

## Abstract

**Background:**

The herbal medicine *Bryophyllum pinnatum* has been used as a tocolytic agent in anthroposophic medicine and, recently, in conventional settings alone or as an add-on medication with tocolytic agents such as atosiban or nifedipine. We wanted to compare the inhibitory effect of atosiban and nifedipine on human myometrial contractility in vitro in the absence and in the presence of *B. pinnatum* press juice (BPJ).

**Methods:**

Myometrium biopsies were collected during elective Caesarean sections. Myometrial strips were placed under tension into an organ bath and allowed to contract spontaneously. Test substances alone and at concentrations known to moderately affect contractility in this setup, or in combination, were added to the organ bath, and contractility was recorded throughout the experiments. Changes in the strength (measured as area under the curve (AUC) and amplitude) and frequency of contractions after the addition of all test substances were determined. Cell viability assays were performed with the human myometrium hTERT-C3 and PHM1–41 cell lines.

**Results:**

BPJ (2.5 μg/mL), atosiban (0.27 μg/mL), and nifedipine (3 ng/mL), moderately reduced the strength of spontaneous myometrium contractions. When BPJ was added together with atosiban or nifedipine, inhibition of contraction strength was significantly higher than with the tocolytics alone (*p* = 0.03 and *p* < 0.001, respectively). In the case of AUC, BPJ plus atosiban promoted a decrease to 48.8 ± 6.3% of initial, whereas BPJ and atosiban alone lowered it to 70.9 ± 4.7% and to 80.9 ± 4.1% of initial, respectively. Also in the case of AUC, BPJ plus nifedipine promoted a decrease to 39.9 ± 4.6% of initial, at the same time that BPJ and nifedipine alone lowered it to 78.9 ± 3.8% and 71.0 ± 3.4% of initial. Amplitude data supported those AUC data. The inhibitory effects of BPJ plus atosiban and of BPJ plus nifedipine on contractions strength were concentration-dependent. None of the test substances, alone or in combination, decreased myometrial cell viability.

**Conclusions:**

BPJ enhances the inhibitory effect of atosiban and nifedipine on the strength of myometrial contractions, without affecting myometrium tissue or cell viability. The combination treatment of BPJ with atosiban or nifedipine has therapeutic potential.

## Background

Preterm birth, defined as birth before 37 weeks of pregnancy, affects 5 to 18% of pregnancies. It is the number one cause of neonatal deaths, and the second leading cause of childhood death under 5 years of age [1–3]. Delaying preterm delivery frequently involves pharmacological inhibition of myometrial contractions (tocolysis) [4]. The main aim of a tocolytic treatment is to delay delivery long enough (24–48 h) for corticosteroid administration to the mother to achieve foetal lung maturation, and for transfer of the mother to a perinatal centre [4, 5]. Several types of tocolytics are currently in use [4, 6, 7]. Atosiban, a competitive oxytocin-vasopressin antagonist [8], and nifedipine, a calcium channel-blocking agent [9], are often given as first tocolytic treatment since they have good efficacy to side-effect ratios [10, 11]. A recent randomised study showed that atosiban and nifedipine resulted in similar perinatal outcomes [12]. Long term (> 1 week) tocolysis is seldom pursued and rarely achieved even though pregnancy prolongation favours perinatal outcomes [13].

*Bryophyllum pinnatum* (Lam.) Oken [syn. *Kalanchoe pinnata* (Lam.) Pers.; family Crassulaceae] is a perennial succulent plant that grows widely in tropical and subtropical areas. In Europe, *B. pinnatum* started to be used in anthroposophic medicine [14] as treatment of preterm labour [15, 16]. Retrospective analyses of the clinical practice revealed good efficacy of *B. pinnatum* preparations in this indication [15, 16], which was corroborated by a matched pair study [17]. Results of a prospective randomised trial on acute tocolysis are in line with good efficacy, but have to be interpreted with caution since - due to poor recruitment rate - the study was discontinued before completion [18]. All clinical studies demonstrated a very good tolerability of *B. pinnatum*. The use of such preparations in the treatment of pre-term contractions was supported by in vitro studies [19, 20].

An assessment of the internal treatment recommendations in the main Swiss obstetrics centres showed that *B. pinnatum* preparations are being prescribed for the treatment of preterm contractions [21]. This is in line with a Swiss online survey which showed that in approximately ¾ of the cases *B. pinnatum* preparations are administered in combination with synthetic tocolytics [22]. Comparable results were obtained in a retrospective analysis of the clinical practice at the University Hospital Zurich [23]. It is still not clear how *B. pinnatum* preparations influence the effects of tocolytics on myometrial contractility. We here compare the inhibitory effect of atosiban and nifedipine on human myometrial contractility in vitro in the absence and in the presence of *B. pinnatum* press juice (BPJ). Given a potentially synergistic effect of these substances, the question is of clinical interest.

## Methods

### Test substances

*B. pinnatum* leaves were harvested on the 25 March 2014 from *B. pinnatum* plants cultivated at the Medical Plants Garden located in S. Roque, Brazil, and that descend from seedlings brought from Weleda AG, Arlesheim, Switzerland, in the past. Plant collection did not affect Brazilian biodiversity and was done in accordance to Brazilian Environmental and Biodiversity laws, mainly Provisional Measure 2186–16 from 23 August 2001 that rules access to genetic resources and traditional knowledge. The Medical Plants Garden from S. Roque belongs to Weleda Brazil and the harvested *B. pinnatum* plants were identified by the Weleda employees Moacyr Copani and Paulo Copani. A voucher specimen ZSS 29717 was deposited at the Zurich Succulent Plant Collection. Leaves were sent by airmail to Weleda Arlesheim, Switzerland, in a refrigerated box. BPJ was obtained by mechanical pressing in a roller, the procedure used in the first step of the production of the active ingredient of Weleda *Bryophyllum* 50% chewable tablets (Weleda AG, Arlesheim). Unfiltered press juice was kept at − 80 °C until use.

Atosiban (Tractocile®, 7.5 mg/mL injectable solution), was purchased from Ferring Pharmaceuticals, Baar, Switzerland. Nifedipine was obtained from Sigma-Aldrich (purity ≥98%, N7634-1G); a 3.7 μg/mL stock solution was prepared in DMSO.

### Design

The ethics committee of canton Zurich approved the study with human myometrium biopsies (KEK-ZH-Nr. 2014–0717, approval date 12.05.2015). Patients were asked prior to elective caesarean sections to donate a myometrium biopsy if the following inclusion criteria were fulfilled: single pregnancy, planned first caesarean section, negative HIV test, age > 18 years, and no tocolysis within 2 weeks before caesarean section.

A myometrial biopsy of approx. 5 g was taken from each study participant at the cranial margin of the uterotomy. The myometrial biopsy was immediately stored in Ringer solution and transported to the lab. Longitudinal strips of muscle of about 15 × 2 × 1 mm were cut and mounted in a myograph bath chamber. Each of the four myograph chambers contained 6 mL of Krebs solution (in mM: NaCl 118, NaHCO_3_ 24.9, KCl 4.7, KH_2_PO_4_ 1.24, CaCl_2_ 2.48, MgSO_4_ 1.21, Glucose 10, EDTA 0.034; pH = 7.4), with temperature regulated at 37 °C and bubbled with 95% O_2_ and 5% CO_2_ (PanGas, Dagmersellen, Switzerland). Contractions were recorded with a DMT800MS myograph (Danish Mayo Technology, Denmark) and transferred to a personal computer via a transducer (ADInstruments PowerLab 4/30). Myometrial strips were allowed to contract spontaneously (which took in most cases approximately 2 h). During this time, the Krebs solution was replaced every 30 min.

In preliminary experiments, concentrations of atosiban, nifedipine and BPJ were determined that would lead to moderate lowering (by 20–30%) of contraction strength. These concentrations were used in the main combination experiments, performed as described below. In preliminary experiments, two slightly different experimental protocols appeared equally promising. We used both protocols, one for the experiments with atosiban, and the second for nifedipine, to find out whether one of the protocols would result in lower standard error of the means (SEM) values.

### Effect of the combination of BPJ with atosiban or with nifedipine on myometrial contractility

In all cases, regular spontaneous myometrial contractions in amplitude and frequency were recorded for 30 min.

When the effects of the combination of BPJ with atosiban were being studied, each one of four strips was treated with one test substance, and contractility was recorded for additional 30 min. Test substances were: Krebs solution, 5 μL (control; *n* = 11); BPJ, 15 μL (0.25% final concentration, corresponding to 2.5 μg/mL; *n* = 13); atosiban, 4.3 μL of 375 μg/mL (0.27 μg/mL final concentration; *n* = 11); and BPJ and atosiban combined (same concentrations, *n* = 12). Temporal and vehicle controls were run in parallel in each experiment to access the decay in contractility of the myometrium with time.

**Fig. 1:**
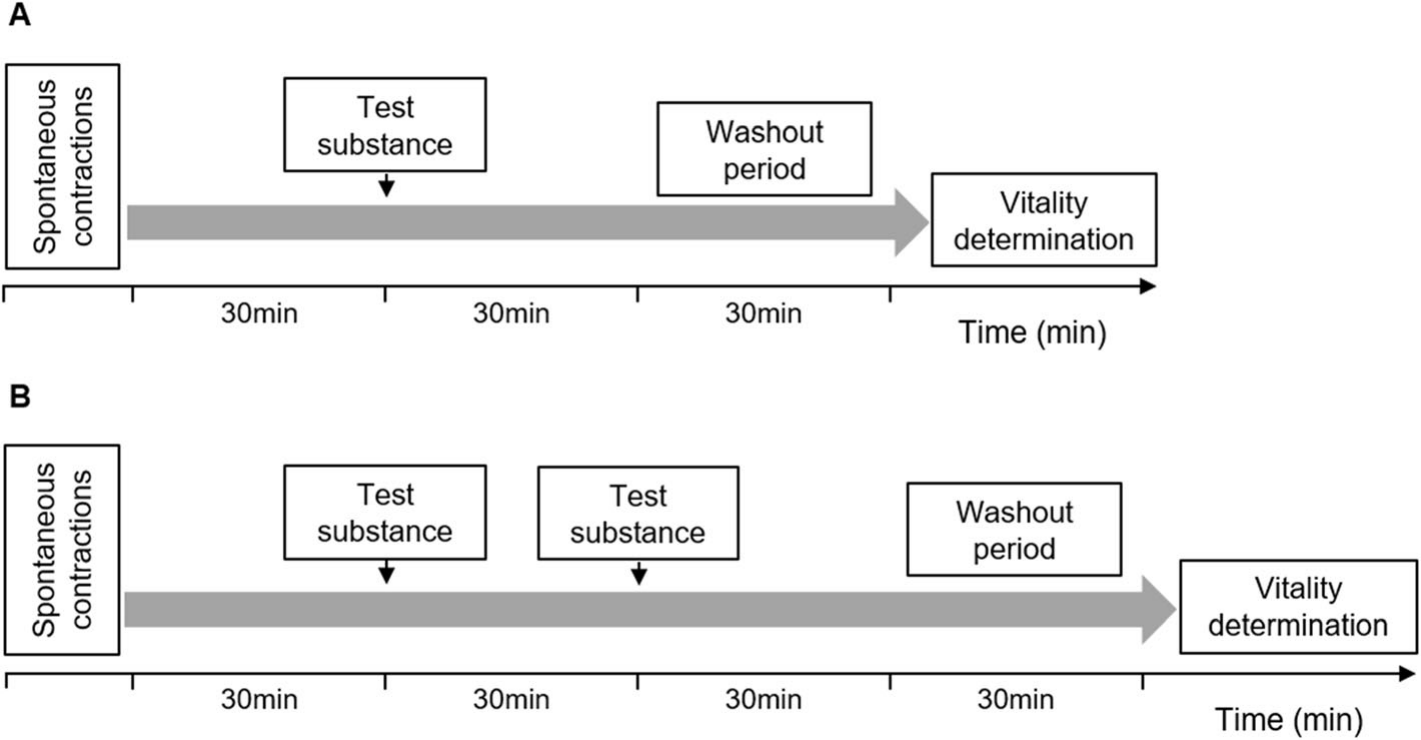
Experimental design for measurement of myometrial contractions. Test substances were added to the organ bath when myometrium strips had been contracting regularly for 30 min. When the effects of BPJ and/or atosiban were being studied (**a**)**,** Krebs solution (control), BPJ, atosiban, or the combination of BPJ and atosiban were added, and contractility was recorded for 30 min. When the effects of BPJ and/or nifedipine were being studied (**b**)**,** Krebs solution (control; two strips) or nifedipine (two strips) was added, contractility was recorded for 30 min, and then BPJ was added to all chambers. Exposure to test substances was followed by a 30 min washout step, with change of Krebs solution at 5, 10, 20 and 30 min

To study the combination of BPJ with nifedipine, Krebs solution, 5 μL (control; n = 13) or nifedipine, 5 μL of 3.7 μg/mL (final concentration 3 ng/mL; n = 11) was added to two chambers each, contractility was recorded for 30 min and thereafter 15 μL of BPJ (final concentration of 0.25 μg/mL; *n* = 10) was added to all four chambers. This resulted in two chambers with BPJ alone and two chambers with the combination of nifedipine with BPJ. After BPJ addition, contractions were recorded for 30 min (Fig. 1b).

### Dose-dependency effect of combination treatments on myometrial contractility

To find out whether the effect of the combination treatments would further increase at higher concentrations, a previously described approach was followed [24]. In brief, when spontaneous contractions were regular for 20 min, Krebs solution was added (addition 0), and contractility was recorded for 20 min. Then, each strip was treated with one test substance by adding 4 times, at time intervals of 20 min, the same volume of a stock solution. Test solutions included: control, 5 μL Krebs solution; BPJ, 15 μL; combination of BPJ (15 μL) plus atosiban (4.3 μL of 375 μg/mL) or BPJ (15 μL) plus nifedipine (5 μL of 3.7 μg/mL). For each substance tested, 5 different biopsies were used (*n* = 5).

### Vitality of myometrial strips

The exposure to the different test substances was followed by a 30-min washing period where Krebs solution was changed several times (at 5, 10, 20 and 30 min). Vitality of the strips was determined at the end of the experiment (30 min after washing) by observation of spontaneous contractions. In all cases, strips were contracting and data were included in the present analysis.

### Myograph data processing

Myometrium contractions were recorded by LabChart Pro 8.0.6 (ADInstruments, Germany) and analysed with the peak analysis module. For each contraction, the area under the curve (AUC) and the amplitude were analysed. Depending on the type of experiments, for each 20 or 30 min interval, the average AUC and average amplitude were calculated, and the number of contractions was noted (frequency). Initial values of AUC, amplitude and frequency of spontaneous contractions (before any addition) were set at 100%. Effects after addition of test substances were expressed as percentage of initial. When studying the combination of BPJ and atosiban and in all dose-dependency experiments, the values obtained in one strip per biopsy were used for further statistical analyses. When investigating the combination of BPJ and nifedipine, two strips per biopsy were used to determine the effect of each substance (BPJ, nifedipine, BPJ plus nifedipine, and control). In this case, average values of the two determinations were calculated and used for further statistical analyses.

### Viability assays in myometrium cell lines

Human myometrial telomerase reverse transcriptase cell line (hTERT-C3) [25, 26], provided by M. Grãos (University of Coimbra, Portugal), was cultured in an 1:1 mixture of DMEM and F-12 supplemented with antibiotics (100 U/mL penicillin and 100 μg/mL streptomycin) and 10% (v/v) heat-inactivated foetal bovine serum (FBS) (all from Gibco, Paisley, UK). Human uterine myometrium smooth muscle cells (PHM1–41), obtained from American Type Culture Collection (ATCC® CRL-3046™) were maintained in ATCC-formulated DMEM (ATCC® No. 30–2002) supplemented with 0.1 mg/mL G-418 (Carl Roth, Zurich, Switzerland), 2 mM glutamine and 10% (v/v) heat-inactivated FBS.

hTERT-C3 cells were seeded at a density of 5 × 10^4^ cells/mL (5 × 10^3^ cells per well) and PHM1–41 cells at a density of 8 × 10^4^ cells/mL (8 × 10^3^ cells per well) into transparent 96-well microplates. 1 day after seeding, cells were exposed to BPJ (2.5–10.0 μg/mL), atosiban (0.27–1.08 μg/mL), nifedipine (3.0–12.0 ng/mL) or the combinations BPJ plus atosiban or BPJ plus nifedipine for 24 h. After exposure, resazurin (Alamar Blue, Invitrogen, Illkirch Cedex, France) was added to cells (final concentration 1.0 mg/mL), and the plate incubated at 37 °C for 4 h. The extent of resazurin reduction was measured in a microplate reader (SpectraMax Paradigm, Molecular Devices, Berkshire, UK) at 570 and 600 nm. For each substance tested, 4 independent experiments were carried out in quadruplicate. Ethyl methanesulfonate (30 mM) [27] and Triton X-100 (1%) were used as a positive control. In each experiment, wells with no test substance added to the culture medium served as untreated control (100% viability). Cell viability was determined according to the following equation:

$$ \mathrm{Viability}=\frac{\left({\mathrm{A}}_{570}-{\mathrm{A}}_{600}\right)\mathrm{sample}-\left({\mathrm{A}}_{570}-{\mathrm{A}}_{600}\right)\mathrm{blank}}{\left({\mathrm{A}}_{570}-{\mathrm{A}}_{600}\right)\mathrm{control}-\left({\mathrm{A}}_{570}-{\mathrm{A}}_{600}\right)\mathrm{blank}} $$

### Cell morphology analysis

Myometrium hTERT-C3 cells and PHM1–41 cells were stained with fluorescent probes for nuclei (double-stranded DNA) and cytoplasm (F-actin), as follows. After treatment with test substances, alone or combined, for 24 h, cells were washed with phosphate buffered saline (PBS; Gibco, Paisley, UK) and fixed with 4% paraformaldehyde (PFA; from Artechemis, Zoffingen, Switzerland) in PBS for 20 min. Cells were then permeabilised with 0.3% Triton X-100 (Sigma, St. Louis, USA) in 1% bovine serum albumin (BSA; Sigma, St. Louis, USA) for 30 min. Then, cells were incubated with a 1:10000 dilution of 4′,6-diamidino-2-phenylindole (DAPI; Sigma, St. Louis, USA) and 1:400 rhodamine phalloidin (Invitrogen, Illkirch Cedex, France) prepared in 0.1% Triton X-100 (Sigma, St. Louis, USA) in 1% BSA, for 4 h, in the dark. Cells were rinsed with PBS and examined with the Leica CTR 6000 microscope (Leica microsystems, Heerbrugg, Switzerland). The entire procedure was performed at room temperature.

### Statistical analyses

Statistical analyses were performed using GraphPad Prism 7 (GraphPad Software, Inc., CA, USA). In all cases, a significance level of *p* < 0.05 was considered statistically significant.

Because of the slightly different experimental set-ups used to investigate the combinations of BPJ with atosiban and of BPJ with nifedipine in the myograph model, different tests were used in the two cases. Data from the combination of BPJ and atosiban measurements were analysed with the Kruskal Wallis test followed by Dunn’s multiple comparisons test. Data from the combination of BPJ and nifedipine were analysed with the Wilcoxon test to compare control with BPJ and nifedipine with the combination (determinations in the same strips, paired test), and with the Mann-Whitney to compare control with nifedipine and with combination (determinations in different strips, unpaired test). Effects of combination treatments on myometrial contractility are expressed in scatter dot plots as mean values ± SEM.

For each test substance, dose-dependency data obtained in the myograph model and cell viability data was analysed with the paired, non-parametric Friedman test. Statistical analyses of cell viability data was followed by Dunn’s multiple comparisons test. In the case of the single concentrations of positive controls used in cell viability assays, the paired t-test was used to compare their effects with untreated control. Myograph measurements on dose-dependency and cell viability data are given as mean ± SEM.

## Results

### Effect of BPJ and atosiban on human myometrial contractility

**Fig. 2:**
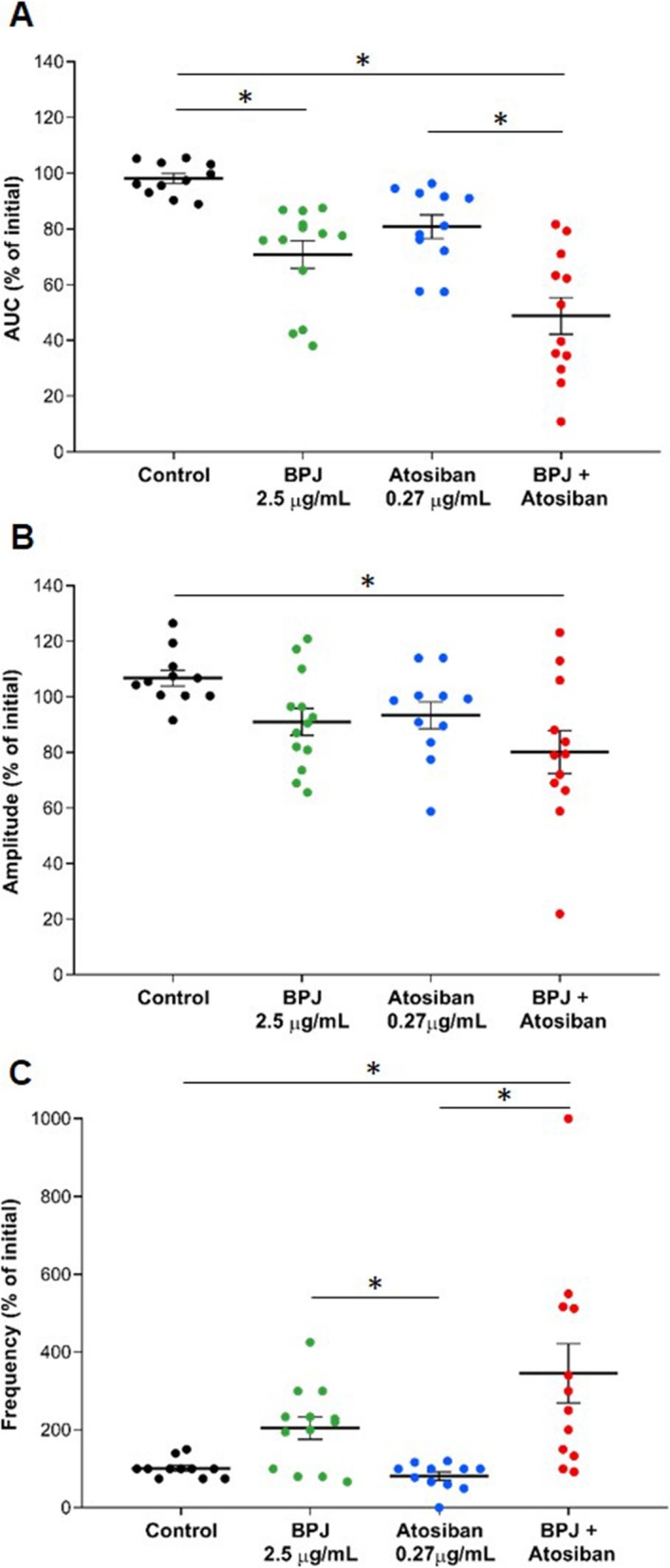
Effect of BPJ, atosiban, and the combination of BPJ with atosiban on human myometrial contractility in vitro. BPJ (green; 15 μL), atosiban (blue; 4.3 μL of 375 μg/mL) or their combination (red, same concentrations) were added to the myograph chamber. The scatter dot plot shows the AUC (**a**), the amplitude (**b**), and the frequency (**c**) of contractions expressed as percentage of initial. Krebs solution was used as negative control (black, 5 μL). Data were obtained from 11 to 15 different biopsies (*n* = 11–15) and are presented as mean value ± SEM. *p < 0.05

The exposure of contracting strips to 2.5 μg/mL BPJ and 0.27 μg/mL atosiban (final concentrations in the bath) led to a decrease of contraction strength (AUC and amplitude; Fig. 2a and b). BPJ alone lowered the AUC to 70.9 ± 4.7% of initial, which was significantly different from the control (*p* = 0.001), while 0.27 μg/mL of atosiban lowered AUC to 80.9 ± 4.1% of initial. When the combination of BPJ and atosiban was added to the organ bath, the AUC decreased to 48.8 ± 6.3%, a value significantly different from control and atosiban alone (*p* < 0.001 and *p* = 0.03, respectively; Fig. 2a).

BPJ decreased the amplitude to 91.0 ± 4.6% of initial, and atosiban to 93.4 ± 4.6% of initial, but the decreases were not statistically significant. The combination of BPJ and atosiban led to a significantly stronger decrease of amplitude (80.1 ± 7.4% of initial, *p* = 0.01; Fig. 2b) relative to control.

The frequency of myometrial contractions increased with the addition of BPJ to 204.7 ± 27.8% of initial, which was significantly higher than with atosiban (81.0 ± 10.1% of initial; *p* = 0.03). Also, the combination of BPJ and atosiban led to a significant increase of frequency (345.4 ± 73.0% of initial) relative to control and atosiban alone (*p* = 0.010 and *p* < 0.001, respectively; Fig. 2c) that per se did not increase frequency.

**Fig. 3:**
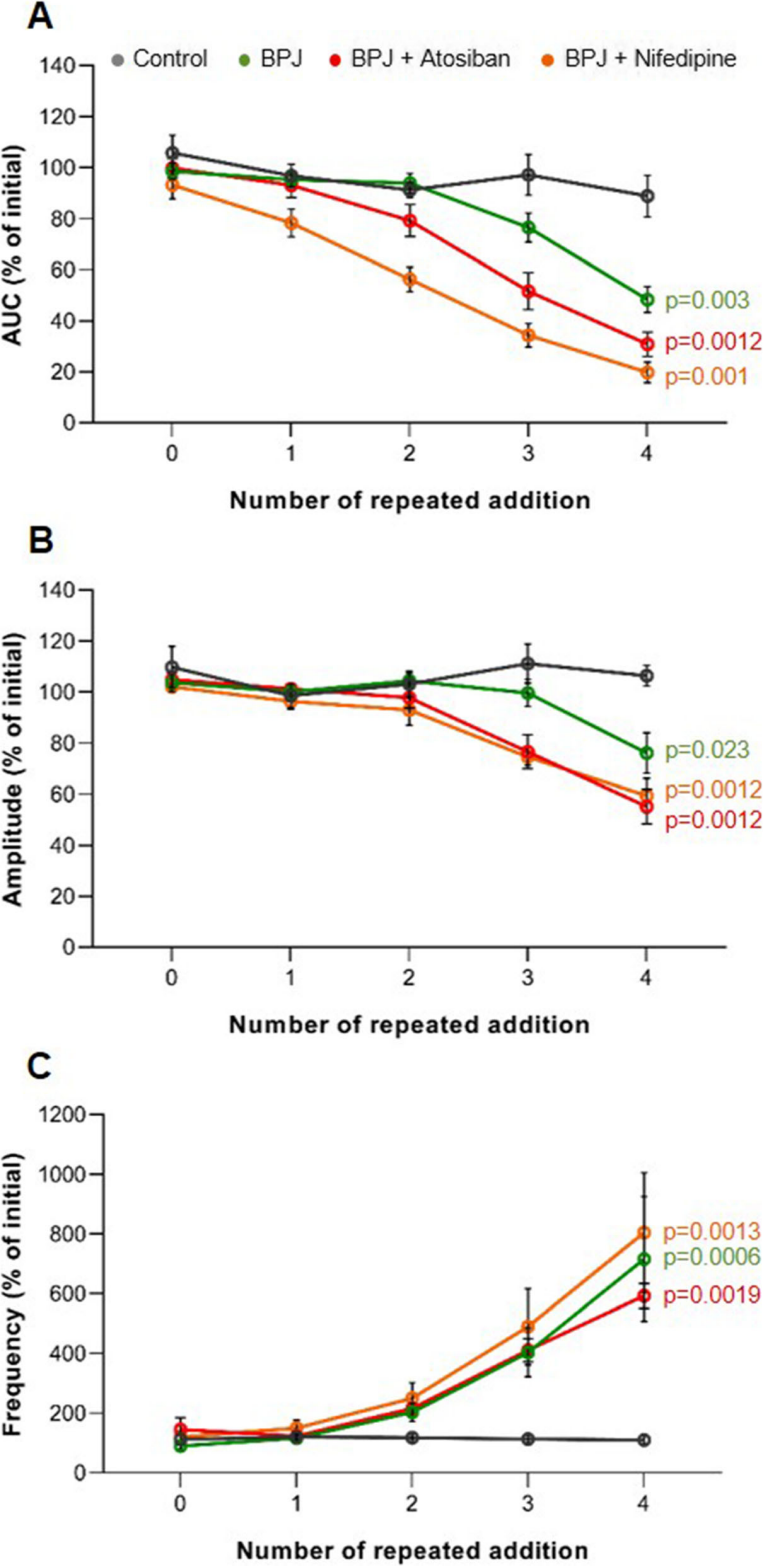
Effect of repeated addition of BPJ plus atosiban (15 μL and 4.3 μL of 375 μg/mL, respectively) and of BPJ plus nifedipine (15 μL and 5 μL of 3.7 μg/mL, respectively) on human myometrial contractility in vitro. All test substances were repeatedly added to the myograph chamber. The line chart shows the AUC (**a**), the amplitude (**b**), and the frequency (**c**). Data were obtained with 5 different biopsies (*n* = 5) and are expressed as percentage of initial. The repeated addition of BPJ was performed for comparison; Krebs solution (5 μL) was used as control. *p < 0.05

Stepwise increase of BPJ and atosiban concentrations led to successive decreases of myometrial contractility strength (Fig. 3a and b).

### Effect of BPJ and nifedipine on myometrial contractility

**Fig. 4:**
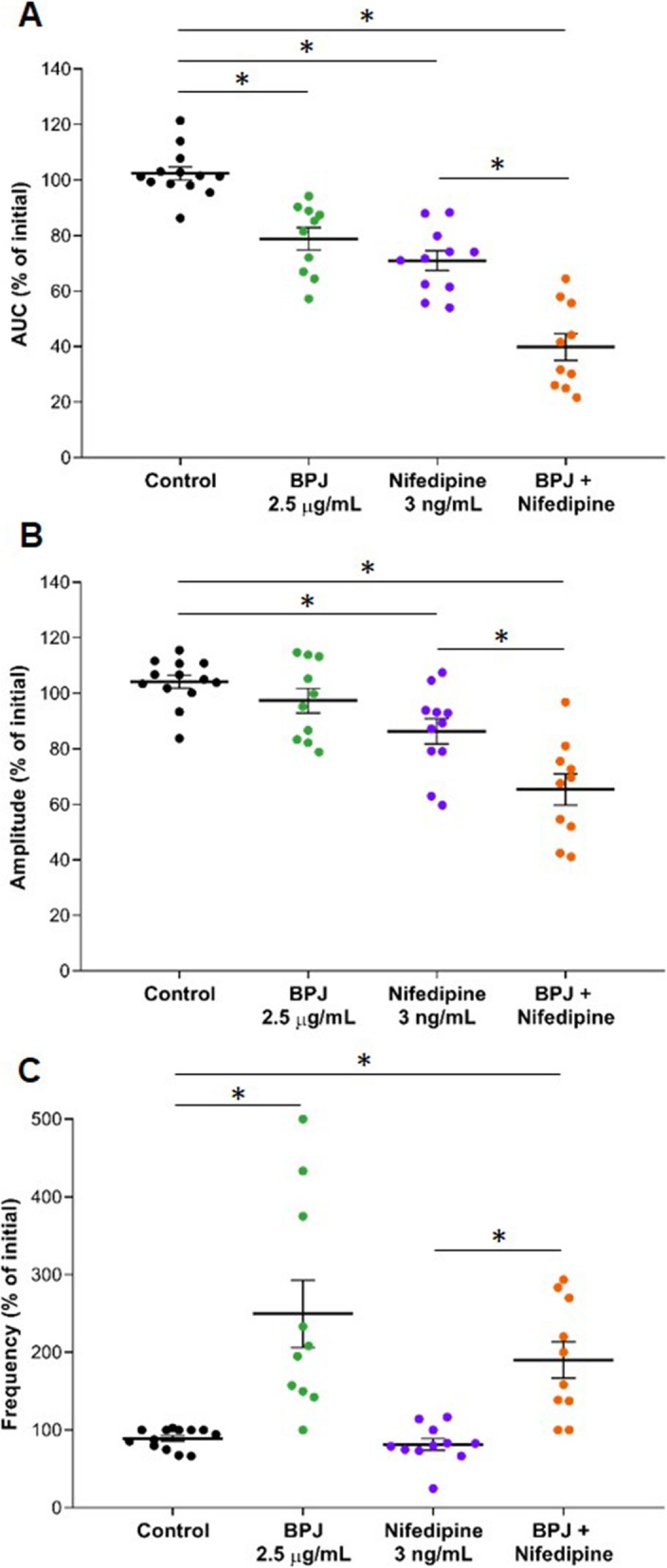
Effect of BPJ, nifedipine, and the combination of BPJ with nifedipine on human myometrial contractility in vitro. BPJ (green; 15 μL), nifedipine (violet; 5 μL of 3.7 μg/mL), or their combination (orange, same concentrations) were added to the myograph chamber. The scatter dot plot shows the AUC (**a**), the amplitude (**b**), and the frequency (**c**) expressed as percentage of initial. Krebs solution was used as negative control (black, 5 μL). Data were obtained from 11 to 13 different biopsies (n = 11–13) and are presented as mean value ± SEM. *p < 0.05

BPJ, nifedipine, or the combination of the two led to a significant decrease of contractions relative to control (Fig. 4a). BPJ alone lead to a decrease to 78.9 ± 3.8% of initial (*p* = 0.003), and nifedipine decreased the AUC to 71.0 ± 3.4% of initial (*p* < 0.001). The combination of nifedipine with BPJ had the strongest effect, as the AUC of contractions was lowered to 39.9 ± 4.6% of initial, which was significantly different from the effect of nifedipine alone (*p* < 0.001).

The amplitude of myometrial contractions decreased with BPJ (91.7 ± 4.7%), nifedipine (86.4 ± 4.4%), and the combination (65.4 ± 5.3%). Compared to control, the effect of nifedipine (*p* < 0.001) and the combination of BPJ with nifedipine (*p* = 0.003) was significant. The combination of BPJ and nifedipine also significantly decreased the amplitude when compared to nifedipine alone (*p* = 0.002; Fig. 4b).

As shown in Fig. 4c, BPJ strongly increased the frequency of myometrial contractions to 257.1 ± 40.6% of initial (*p* < 0.001). In contrast, nifedipine alone had no effect on frequency when compared to control. However, the combination of BPJ and nifedipine led to an increase of 190.1 ± 22.2% of initial, which was significantly different from control (*p* < 0.0001) and from nifedipine alone (*p* < 0.001; Fig. 4c).

Exposing the strips to successively higher concentrations of the combination of BPJ and nifedipine led to stepwise increases of the inhibitory effects on myometrial contractility (Fig. 3a and b, data on BPJ + Nifedipine).

### Effects on myometrial viability

**Fig. 5:**
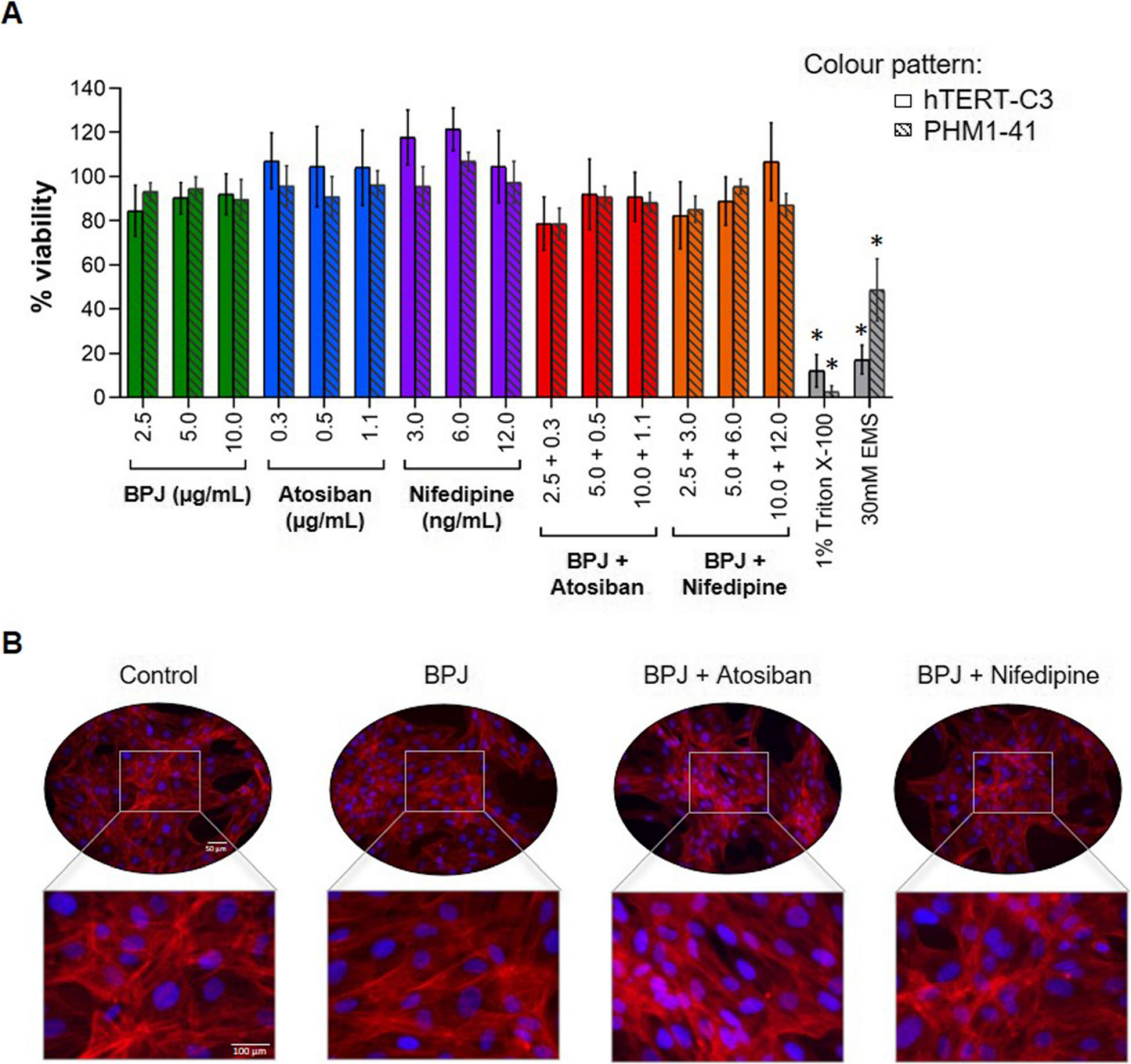
Effect of BPJ, atosiban, nifedipine, BPJ plus atosiban and BPJ plus nifedipine on myometrium cell viability. (**a**) Cell viability assays were performed in the presence of BPJ (2.5–10.0 μg/mL), atosiban (0.3–1.1 μg/mL) and nifedipine (3.0–12.0 ng/mL), as well as of BPJ plus atosiban and BPJ plus nifedipine (same concentrations as with single treatments) using hTERT-C3 and PHM1–41 human myometrium cell lines. Cells were incubated with the test substances for 24 h. Triton X-100 (1%) and ethyl methanesulfonate (30 mM) were used as positive controls. Data is presented as mean ± SEM of 4 independent experiments (*n* = 4), each carried out in quadruplicate; *p < 0.05. (**b**) Staining of nuclei (blue) and actin (red) from hTERT-C3 cells untreated (i.e. control) or upon treatment with BPJ (10.0 μg/mL), BPJ plus atosiban (10.0 μg/mL and 1.1 μg/mL, respectively), and BPJ plus nifedipine (10.0 μg/mL and 12.0 ng/mL, respectively). The images are representative of four independent cultures

Under our experimental conditions, myometrium strips were still contracting spontaneously after the washing step at the end of the myograph experiments, revealing that the test substances (single or in combinations) were not toxic to myometrial tissue (data not shown). To assess the cytotoxicity of the test substances using a different read-out, viability experiments were performed with two human myometrial cell lines (hTERT-C3 and PHM1–41). The test substances, alone or in combination, were not cytotoxic at similar or even higher concentrations than those used in the main combinations experiment, and at a markedly longer exposure time (24 h; Fig. 5a). At the end of cell viability experiments, cell morphology was evaluated by fluorescence microscopy. Visual examination revealed that the test substances did not affect the morphology of myometrial cells. In particular, the intact nucleus morphology of hTERT-C3 cells (Fig. 5b) or PHM1–41 cells (data not shown) treated with the highest concentrations of the various test substances reveals that the cells were not undergoing apoptosis.

## Discussion

Press juice from *B. pinnatum* leaves, the active ingredient of chewable tablets that are being used in the management of preterm labour in Switzerland, enhances the inhibitory effect of the oxytocin receptor antagonist atosiban on contraction strength in human myometrium strips. BPJ also enhances the effect of nifedipine, a voltage-dependent calcium channel blocker that is in off-label use as a tocolytic. Both the combination BPJ with atosiban and BPJ with nifedipine show dose-dependent effects on human myometrial contractility. Reduced viability of myometrial tissue or cells does not play a role in the observed results. Taken together, our data corroborate the clinical use of these combination treatments.

The main strength of our study is the use of the physiologically most relevant model to study the process of myometrial contractions in human material, with the possibility to compare the effect of different substances, alone and in combination. In fact, our study depicts a proof of principle for the therapeutic potential of the combination of standard tocolytic medications with BPJ. Alternatives would be animal models, but the process of labour differs between humans and animal species [28, 29]. Limitations of our model are the availability of myometrium biopsies and the very low throughput that can be achieved with an organ bath model. For these reasons, the number of tested concentrations had to be kept low. Also due to the low throughput, it would be advantageous to further reduce the variability of results (and therefore of needed experiments) in future projects. For this reason, two variant experimental protocols were used in this work. The comparable SEM values obtained indicated that the two protocols were equally suited.

Several signalling pathways are known to increase intracellular calcium concentrations leading to contraction of myometrium cells. Some of these pathways are triggered by binding of oxytocin to the corresponding G-protein coupled receptor, but membrane depolarisation with concomitant opening of voltage-gated calcium channels may also play a role [30]. The two tocolytics used in the present study prevent the increase in intracellular calcium concentration by different mechanisms, namely by blocking oxytocin receptors in the case of atosiban, or by blocking voltage-gated calcium channels in case of nifedipine [11]. Previous work showed that BPJ inhibits oxytocin-induced increase of intracellular calcium concentration in myometrium cells. BPJ did not prevent, but delayed the depolarisation-induced increase of intracellular calcium in cells with voltage-gated channels [31]. In myometrial strips, the combination of atosiban with calcium-channel blockers (nicardipine or nifedipine) led to additive inhibitory effects, indicating that simultaneous targeting of these two pathways has clinical potential [32, 33]. Therefore, it appears conceivable that the delaying effect of BPJ on depolarisation-induced increase of intracellular calcium can enhance the effects of atosiban. Likewise, the inhibition of oxytocin-induced increase of intracellular calcium concentration by BPJ should synergise with the effects of nifedipine. To which extent inhibition by BPJ of each of these two signalling pathways contributes to the observed in vitro results or to the effectiveness of *B. pinnatum* preparations in the treatment of preterm labour is currently not known.

BPJ is known to increase the contraction frequency of myometrium strips [19, 20] and this was also observed in the combination with atosiban or nifedipine (Figs. 2, 3 and 4). Our previous work suggests that various components of *B. pinnatum* leaves might contribute to increase contraction frequency of myometrium strips [24]. Both a bufadienolide-enriched fraction and a flavonoid-enriched fraction (but not the corresponding flavonoid aglycon mixture) seemed to contribute to the frequency increase. Whether the signalling pathways activated by BPJ might trigger a partial membrane-depolarisation reserves further investigations. Trying to translate the increase in frequency seen in myometrium strips into the clinical situation, we feel tempted to suggest that *B. pinnatum* preparations – but not other tested tocolytics – could induce a type of conversion of labour-contractions into other, high frequency and painless, and most importantly not-effective, contractions. These are well known in the praxis, and often interpreted as myometrium training uterine contractions that do not lead to labour. In retrospective studies on the tolerability of *B. pinnatum*, no clinically relevant increases of contraction frequency have been observed, neither when used as single treatment nor in combination with tocolytics [15–17, 23]. Also, no increase was reported in a prospective observational study which even included women with uterine tachysystoles [22]. On the contrary, a significant lowering of contraction frequency was observed after 4 h of treatment with *B. pinnatum* 50% tablets in a previous randomised trial on acute tocolysis (*n* = 13) [18].

Although the main combination experiments were performed in vitro and with concentrations leading to moderate effects, we compared the concentrations of atosiban and nifedipine used in vitro with the corresponding plasma concentrations during tocolysis. In the case of atosiban, the mean plasma concentration at steady state is 0.44 ± 0.07 μg/mL [34], and in the case of nifedipine is 67.4 ± 28.4 ng/mL [35]. Therefore, whereas the concentration of atosiban in the myograph experiments is rather close to that measured in the plasma, the concentration of nifedipine was markedly lower. Given the lack of pharmacokinetic data with BPJ, it is not possible at this point to compare the concentration in the myograph experiments with known plasma concentrations.

Currently used standard tocolytic treatments are not always able to prolong pregnancy for at least 48 h [7]. At the same time, combinations of standard tocolytics are not recommended by the Swiss Society for Gynaecology and Obstetrics current Swiss guidelines [36], nor by the guidelines from the National Institute for Health and Care Excellence (NICE) [37] because of concerns about side-effects. Our data show that BPJ enhances the inhibitory effect of atosiban and nifedipine on myometrium contractility. In Germany and Switzerland, *B. pinnatum* preparations (containing BJP as the active ingredient) have been used for decades in clinics and private practices of anthroposophic medicine [15, 16, 38]. In Switzerland, *B. pinnatum* preparations are being recommended [21] and used to stop pre-term contractions also in conventional clinical practice, often as an add-on treatment [22, 23]. In the case of atosiban, a combination at low dosages with *B. pinnatum* would lower the overall medication costs. As for nifedipine, a lowering of the dosage in a combination with *B. pinnatum* would have the advantage of limiting the well-known cardiovascular side effects, such as palpitations, hypotension, flushes, headache, and gastro-intestinal symptoms like gastric upset and constipation [39].

## Conclusion

We provide here evidence for the potential of drug combinations of atosiban and nifedipine with *B. pinnatum*. Such combinations may lower the required dosage of tocolytics, thereby decreasing treatment costs and reducing maternal and foetal side effects. This could help to reduce early tocolysis failure and to increase the percentage of patients that reach a 48 h delay of delivery. Prospective randomised studies are needed to substantiate such combination treatments.

## Data Availability

Data obtained during the current study are available from the corresponding author on reasonable request. The biological materials used (myometrium strips) are not available since they have to be used fresh.

## Abbreviations

AUC: Area under the curve
BPJ: *Bryophyllum pinnatum* leave press juice
